# METTL3 as a master regulator of translation in cancer: mechanisms and implications

**DOI:** 10.1093/narcan/zcae009

**Published:** 2024-03-05

**Authors:** Margalida Esteva-Socias, Francesca Aguilo

**Affiliations:** Department of Molecular Biology, Umeå University, SE-901 85Umeå, Sweden; Wallenberg Centre for Molecular Medicine, Umeå University, SE-901 85Umeå, Sweden; Department of Molecular Biology, Umeå University, SE-901 85Umeå, Sweden; Wallenberg Centre for Molecular Medicine, Umeå University, SE-901 85Umeå, Sweden

## Abstract

Translational regulation is an important step in the control of gene expression. In cancer cells, the orchestration of both global control of protein synthesis and selective translation of specific mRNAs promote tumor cell survival, angiogenesis, transformation, invasion and metastasis. N6-methyladenosine (m^6^A), the most prevalent mRNA modification in higher eukaryotes, impacts protein translation. Over the past decade, the development of m^6^A mapping tools has facilitated comprehensive functional investigations, revealing the involvement of this chemical mark, together with its writer METTL3, in promoting the translation of both oncogenes and tumor suppressor transcripts, with the impact being context-dependent. This review aims to consolidate our current understanding of how m^6^A and METTL3 shape translation regulation in the realm of cancer biology. In addition, it delves into the role of cytoplasmic METTL3 in protein synthesis, operating independently of its catalytic activity. Ultimately, our goal is to provide critical insights into the interplay between m^6^A, METTL3 and translational regulation in cancer, offering a deeper comprehension of the mechanisms sustaining tumorigenesis.

## Graphical Abstract

**Graphical Abstract:**
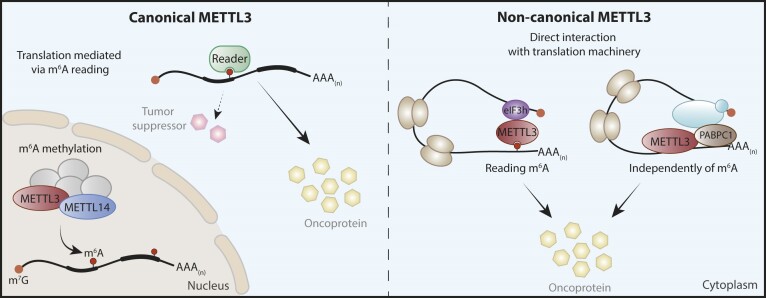

## Introduction

Gene expression regulation is a fundamental aspect of cell biology, orchestrating the complex transformation of genetic information into functional gene products. At both the transcriptional and post-transcriptional levels, a wide variety of mechanisms tightly control gene expression, ranging from chromatin remodeling, transcription, RNA processing, and RNA stability to translation and turnover (1,2). Dysregulations at any of these regulatory steps represent a hallmark of cancer. Particularly, enhanced translation, the most energy-consuming biological process, is essential for cancer cells to sustain proliferation, angiogenesis, and migration (3–5).

**Figure 1.**
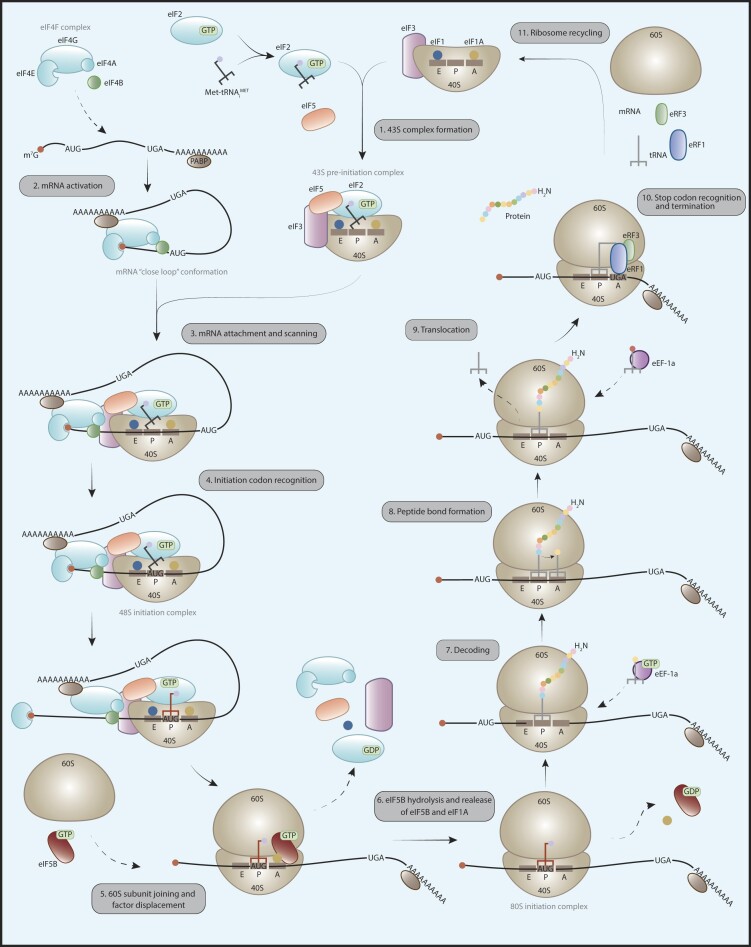
Translation process in eukaryotes. Translation involves four main phases: initiation (steps 1 to 5), elongation (steps 6 to 9), termination (step 10), and recycling (step 11). Eukaryotic translation begins with the assembly of the 43S pre-initiation complex, comprising the 40S ribosomal subunit, eIF2-GTP-Met-tRNAiMET, eIF3, eIF5, eIF1 and eIF1A. The target transcript is activated by the eIF4F complex (eIF4E, eIF4G, eIF4A), eIF4B and PABP, which promotes the loop conformation. This mRNA then binds to the 43S pre-initiation complex, forming the 48S complex, initiating the start codon recognition scan. Upon recognition, the 60S ribosomal subunit and eIF5B-GTP associate, releasing cap-binding factors, eIF2-GDP, eIF1 and eIF5. Elongation involves cycles in which mRNA moves through the ribosome, with tRNAs in the P site carrying the polypeptide, and eEF1α-GTP incorporating new aminoacyl tRNA into the A site. Peptide transfer occurs, followed by ribosome translocation along the mRNA. This cycle repeats until a stop codon is recognized by eRF1 and eRF3, releasing the peptide. This is followed by Rli1/ABCE1 binding, hydrolysis and dissociation of ribosomal subunits. Finally, tRNA, mRNA, and terminator factors are released, resulting in separated ribosomal subunits for new translation cycles.

The translation of messenger RNA (mRNA) into proteins comprises four different stages, initiation, elongation, termination, and recycling (6,7). Translation initiation is the most commonly modulated step, requiring the action of multiple eukaryotic initiation factors (eIFs) such as eIF1, eIF1A, eIF2, eIF3 and eIF4, along with other accessory proteins like PABP (8) (Figure 1). Although our understanding of the basic process of translation, including the roles of canonical factors such as initiation, elongation and termination factors, has advanced considerably, its complexity and context-specific regulation suggest that much remains unknown.

Amongst other factors, chemical modifications of RNA influence all stages of translation. To date, more than 170 chemical modifications are known to be incorporated into all types of RNA (9). The majority of these marks are enzymatically deposited in a site-specific manner affecting RNA stability, structure, RNA–RNA and RNA–protein interactions (10). During translation initiation, RNA modifications may guide the recruitment of ribosomes to the start codon, modulating the translation efficiency (11,12). The presence of RNA modifications also defines the secondary structure of the RNA molecule, having a direct impact on the accessibility to ribosomes and other initiation factors to the start codon (13,14). In addition, modifications deposited on transfer RNAs (tRNAs) and ribosomal RNAs (rRNAs) influence the codon recognition during elongation, ensuring translation accuracy (11,15). At the termination step, they become essential for the stop codon recognition and proper release of the nascent protein (15–17).

**Figure 2.**
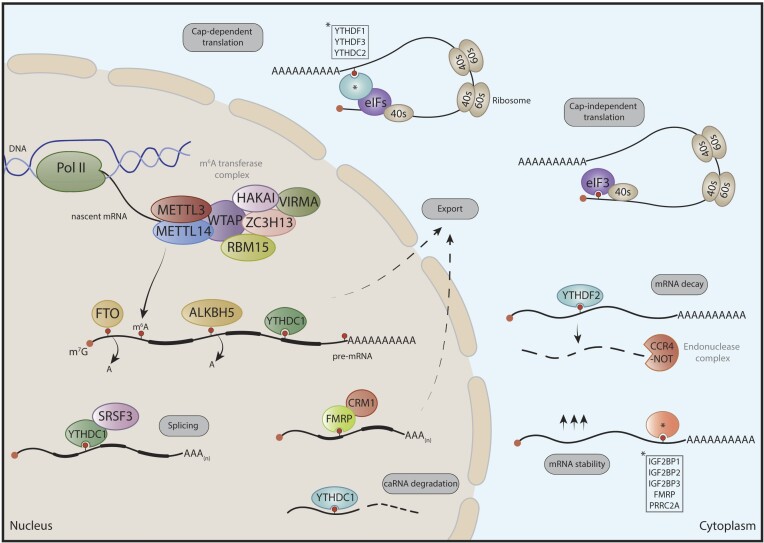
Schematic representation of m^6^A writers, erasers and readers and their impact on mRNA metabolism. m^6^A is deposited by the m^6^A methyltransferase complex, consisting of the heterodimer METTL3-METTL14 and other accessory proteins (WTAP, VIRMA, HAKAI, ZC3H13, RBM15/15B). m^6^A is erased by the demethylases FTO or ALKBH5. In the nucleus, m^6^A regulates mRNA export, splicing and degradation of chromosome-associated RNAs (caRNA). In the cytoplasm m^6^A influences mRNA stability, both cap-dependent and independent translation, and decay. Asterisk (*) indicates that more than one reader protein can mediate a specific mRNA fate.

N6-methyladenosine (m^6^A), the most prevalent internal modification in eukaryotic mRNA, directly impacts translation (18–20). It consists of the addition of a methyl group to adenosine at the N6 position in nascent transcripts by a writer complex, being methyltransferase-like 3 (METTL3) the enzyme responsible for catalyzing the addition of a methyl group (21). This enzymatic activity relies on its association with METTL14, which acts as an RNA-binding scaffold together with other interactors that can control m^6^A deposition in cell-specific ways. As a reversible modification, m^6^A can be removed by the ‘eraser’ proteins Fat Mass and Obesity-Associated protein (FTO) and AlkB Homolog 5 (ALKBH5) (22,23). Once deposited and to exert a function within the cell, the m^6^A mark is recognized by ‘reader’ proteins, which are essentially RNA-binding proteins (RBPs) that dictate the fate of the modified transcript based on their molecular features and cellular localization (Figure 2). Readers of m^6^A include proteins harboring the YT521-B homology (YTH) domain, including YTH domain family 1–3 (YTHDF1-3) and YTH domain containing 1–2 (YTHDC1-2). YTHDF proteins reside in the cytoplasm where YTHDF2 promotes mRNA decay (24), YTHDF1 facilitates translation by interacting with initiation factors and facilitating ribosome loading (25), and YTHDF3 binds to YTHDF1 or YTHDF2 to mediate translation or degradation of m^6^A-modified mRNAs, respectively (26,27). Conversely, YTHDC1 localizes to the nucleus where it regulates alternative splicing, degradation of chromatin-associated RNAs and nuclear export (28–31). The functions of YTHDC2 are still under investigation but it is known to be important for spermatogenesis and oogenesis regulating mRNA stability and translation (32–35). Aside from its m^6^A-binding function, YTHDC2 recognizes U-rich motifs in the 3′UTR, enabling the separation of the mitotic and meiotic transcriptomes and ensuring mouse fertility (36). In addition, insulin-like growth factor 2 mRNA-binding proteins (IGF2BPs), particularly IGF2BP1-3, play a crucial role in mediating mRNA stabilization, thereby finely tuning mRNA half-life and translational kinetics. Fragile X mental retardation protein (FMRP) has been also identified as an m^6^A reader, implicated in synaptic plasticity, mRNA nuclear export, and mRNA stability (37,38). Furthermore, the proline-rich coiled-coil 2A (PRRC2A) has emerged as a novel m^6^A reader involved in the regulation of mRNA stability in oligodendrocyte specification and myelination (39). A comprehensive review of m^6^A readers is available elsewhere (40,41). Interestingly, other RBPs, known as anti-readers, are negatively affected by the m^6^A mark, recognizing the secondary structure induced by m^6^A and repelled by it (42,43). Among the well-described anti-readers of m^6^A are human pumilio 2 (hPUM2), stress granule proteins G3BP1 and G3BP2, RBM42, USP10, CAPRIN1, as well as the RBPs LIN28A and EWSR1 (13,44). Furthermore, m^6^A orchestrates RNA structure-dependent accessibility, leading to the release of specific single-stranded sequences and enabling the binding of indirect m^6^A readers, a mechanism termed ‘m^6^A-switch’ (13,45).

**Figure 3.**
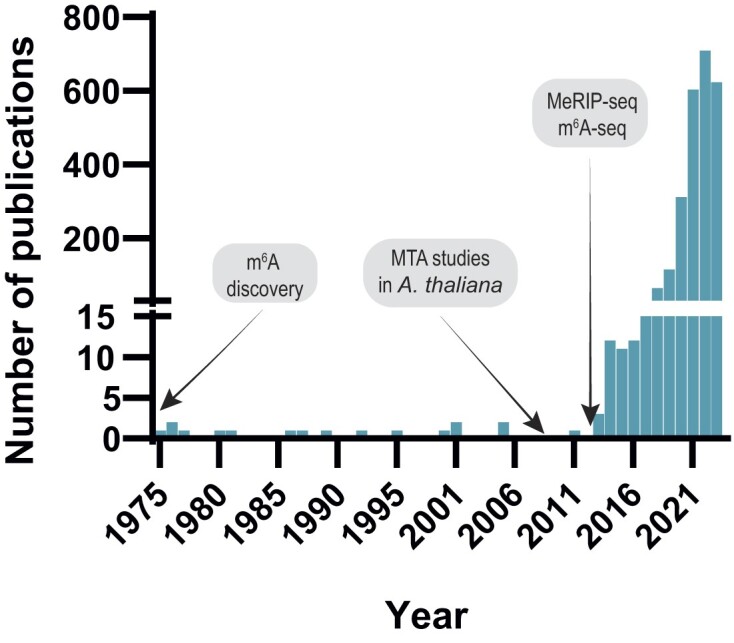
Publication trends on m^6^A and cancer from PubMed. The figure depicts the yearly publication count from a combined Pubmed search using the terms ‘N6-methyladenosine’ or ‘N(6)-methyladenosine’ and ‘cancer’. It highlights significant milestones, including the initial discovery of m^6^A in 1974 the pioneering studies on METTL3 (named MTA or MTA70) in *Arabidopsis thaliana* in 2008, and the development of the first high throughput techniques -MeRIP-seq and m^6^A-seq-enabling m^6^A mapping in 2012.

Although m^6^A was first discovered in the 1970s (46,47), it was not until 1997 that METTL3 (named mRNA adenosine methylase or MTA) was first purified (48). Its role was subsequently demonstrated in 2008 in *Arabidopsis thaliana* (49). In this first pioneering study, the inactivation of MTA in *Arabidopsis* resulted in impaired embryo development, as a consequence of m^6^A methylation deficiency. The authors also reported the interaction between MTA and FIP37, characterized as a plant homolog of WTAP, as essential for alternative splicing (49). In 2012, two independent research groups developed high-throughput methods based on m^6^A immunoprecipitation followed by next-generation sequencing (MeRIP-seq or m^6^A-seq) to study m^6^A transcriptome-wide, and allowing in-depth functional studies (50,51). Since then, understanding the role of m^6^A in pathological processes and disease, such as cancer, has been the focus of numerous studies (Figure 3). Rather than playing a unique role, research has proved that the role of m^6^A and METTL3 in cancer is context-dependent. Their influence on translation regulation can either drive oncogenic processes or act as tumor-suppressing factors depending on the specific transcripts where m^6^A is deposited (52,53).

Interestingly, recent findings have revealed a broader role for METTL3 that extends beyond its conventional methyltransferase function. METTL3 has been shown to translocate to the cytoplasm of cancer cells, where it orchestrates the translation of specific oncogenic transcripts. This adds a layer of complexity to its role in gene expression regulation and its potential impact on cancer biology. In this review, in addition to the m^6^A-dependent mechanisms regulating translation in cancer, we describe the current understanding of the cytoplasmic function of METTL3. Additionally, we illustrate potential molecular mechanisms mediating METTL3 distinct subcellular localization, contributing to execute its unexplored functions.

## Regulation of translation by m^6^A

**Figure 4.**
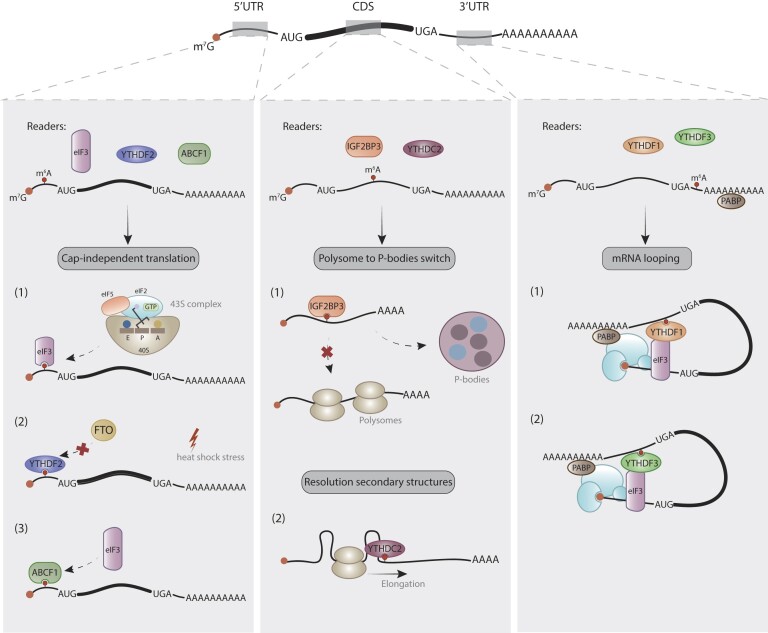
Mechanisms of translational regulation mediated by m^6^A sites in 5′UTR, coding sequences (CDS) and 3′UTR regions. In the 5′UTR, under certain stress conditions, eIF3 can act as a reader and recruit the 43S initiation complex, YTHDF2 protects m^6^A-transcripts from FTO demethylation and ABCF1 can recruit eIF3 and initiate translation. In the CDS, IGF2BP3 can bind to the m^6^A mark and mediate the switch of transcripts from polysomes to P-bodies, whereas YTHDC2 contributes to the resolution of RNA secondary structures and alleviates ribosome stalling. Finally, in the 3′UTR region, m^6^A is mainly recognized by either YTHDF1 or YTHDF3 to mediate the cap-dependent translation by promoting the loop conformation of the RNA.

The specific deposition site of m^6^A within the mRNA has diverse functional implications (Figure 4). In the 3′UTR, m^6^A primarily impacts mRNA stability and turnover, yet several studies suggest it also plays a role in translation. For instance, YTHDF1 recognizes 3′UTR m^6^A modifications and, via mRNA looping, facilitates the interaction with eIF3 to initiate translation (24). This mechanism can also take place in cooperation with YTHDF3 (26,27). Within the CDS, m^6^A influences tRNA selection, translation-elongation dynamics, and codon reading accuracy (54–56). Conversely, although 5′UTRs exhibit minimal m^6^A methylation, several studies propose that m^6^A modifications in the 5′UTRs directly bind to eIF3, promoting translation initiation independently of cap-binding proteins (57). However, recent structural studies have refuted the role of m^6^A at 5′UTRs in translation (58).

It is worth noting that conflicting studies challenge the initial understanding suggesting that YTHDF1 exclusively promotes translation, YTHDF2 facilitates mRNA decay, and YTHDF3 performs both functions (24–27). These studies suggest that YTHDF proteins likely bind the same m^6^A-modified mRNAs, rather than distinct ones, to mediate mRNA degradation (59). Furthermore, they propose that YTHDF1 and YTHDF3 might not trigger translation in HeLa cells. Such redundancy in the readers’ role was also observed during early development in mice and zebrafish (60,61). However, further research aiming to resolve these discrepancies demonstrated that YTHDF1 may play a role in promoting translation, potentially through interactions with different protein partners compared to YTHDF2 (62). The authors re-analyzed a protein localization map generated by Bio-ID from HEK293 cells (63), and found that YTHDF1 tends to localize similarly to cytosolic RNP granules, akin to eIFs, whereas YTHDF2 shows stronger colocalization with CNOTs. YTHDF3 resides in the margin between CNOTs and eIFs. Interestingly, the authors also show that depleting all three YTHDF proteins leads to global stabilization of the whole transcriptome as a result of increased P-formation, yet this effect is not m^6^A dependent (62). Moreover, a single-molecule analysis revealed that individual copies of a given mRNA can be bound by multiple YTHDF proteins during their lifetime, suggesting that the binding of YTHDFs to the target mRNAs may not always promote an immediate degradation of the transcript. This could be due to the inefficient recruitment of the degradation machinery, the weak interaction with the mRNA or different RNA:protein interaction affinities of the different paralogs (64).

Additionally, post-translational modifications (PTMs) could potentially explain the regulatory mechanisms behind the control of various biological functions of the YTHDF readers. Thus, a recent study showed that *O*-GlcNAcylation (O-GlcNAc) regulates YTHDF1 and YTHDF3-mediated translation of m^6^A-modified mRNAs. O-GlcNAc, a reversible and dynamic monosaccharide post-translational modification, prevents the interaction of YTHDF1 and YTHDF3 with translation-associated machinery in a cell cycle-dependent manner. During the M phase, highly *O*-GlcNAcylated YTHDF1 and YTHDF3 correlate with lower translational activity, whereas *O*-GlcNAc modification is reduced in the S phase contributing to high translation rates of m^6^A-modified transcripts (65).

### Regulation of mRNA translation by m^6^A in the 3′UTR

One of the many mechanisms by which m^6^A promotes translation involves the reader YTHDF1, which directly interacts with the translation initiation factors eIF3A and eIF3B, enhancing the translation efficiency of m^6^A-modified mRNAs in mammals (25). YTHDF1 has been found to play a crucial role in translation regulation in various cancers. For instance, in gastric cancer YTHDF1 promotes the translation of the Wnt receptor frizzled7 (FZD7), leading to the hyperactivation of the Wnt/ß-catenin pathway and promoting gastric tumorigenesis (66). Likewise, EIF3C, FOXM1 and RANBP2 are upregulated by this mechanism in ovarian cancer and emerge as critical players in the progression of breast, and cervical tumors, respectively (67–69). EIF3C, a translation initiation factor, is upregulated in several malignancies and has been identified as a therapeutic target for a wide range of cancers (70). FOXM1, an oncogenic transcription factor, orchestrates cell proliferation and cell cycle and contributes to angiogenesis and metastasis (71). RANBP2 is a nucleoporin involved in mitosis and macromolecule transport that has been associated with tumor growth, migration and invasion (72,73). This model proposes that YTHDF1 recruits eIF3a specifically to the 3′UTR region and the stop codon, where YTHDF1 binding sites predominantly reside, most likely through an mRNA looping mechanism (74). YTHDF3 has also been described to promote translation through the same mechanism (26,27). Additionally, YTHDF1 has been demonstrated to regulate the expression of the translation initiation factors eIF3A, eIF3B and eIF3C, enhancing global translation and indicating multiple mechanisms through which YTHDF1 influences translation (75).

### Regulation of mRNA translation by m^6^A in the 5′UTR

Alternatively, m^6^A residues located within the 5′UTR have been shown to mediate *N*^7^-methylguanosine (m^7^G) cap-independent translation. The conventional method of mRNA translation begins with the ribosome binding to the cap structure at the 5′UTR and scanning until a start codon is found. However, under certain stress conditions, the cells adopt alternative mechanisms that include the use of an internal ribosome entry site (IRES) in the 5′UTR to directly recruit the ribosome to the start codon. The original study supporting this idea demonstrated that eIF3 is able to act as a reader directly binding to the m^6^A mark in the 5′UTR, independently of YTHDF1, and recruit the 43S complex to initiate translation (57). Subsequent studies have shown that under heat shock stress, YTHDF2 translocates to the nucleus to bind 5′UTR-m^6^A, protecting the targeted transcript from FTO-mediated demethylation. Such an increase in m^6^A at the 5′UTR allows for cap-independent translation of for example *Hsp70* mRNA (76). In addition, ABCF1 is essential for the direct binding of eIF3, facilitating the translation of mRNAs under normal culture conditions, and supporting the hypothesis of cap-independent translation in the absence of any cellular stress. Interestingly, the same authors demonstrated that METTL3 translation is subject to ABCF1 regulation in a positive feedback loop when cap-dependent translation is inhibited (77).

In contrast, a recent study suggests that the role of m^6^A in the 5′UTR in translation initiation has a marginal effect on translation yield, kinetics of translation initiation complex assembly, or start codon recognition under both homeostasis and oxidative stress (58). In particular, the authors show by structural analysis that the m^6^A mark does not affect the conformation of the m^6^A purine ring. Moreover, its thermodynamic contribution to the translation initiation factor eIF2α is notably lower compared to the contribution of the m^7^G cap structure to eIF4E, suggesting that the major energy gain is driven by the cap modification rather than m^6^A (58). Notably, the authors focused on the effect of a single m^6^A mark in the 5′UTR region in close proximity to the start codon. To better understand the role of m^6^A in influencing translation across the 5′UTR region, future investigations should address the possible cumulative effect of m^6^A clusters and the effect of these clusters in distant regions.

### Regulation of mRNA translation by m^6^A in the CDS

Around 35% of m^6^A sites are found within the coding region (CDS) of transcripts (78). Nevertheless, the specific mechanism and its impact on translation is still controversial. For instance, in HEK293T cells, m^6^A deposition within the CDS strongly inhibits translation, especially when occurring in the first codon (79). Likewise, higher levels of m^6^A in the CDS of mouse embryonic fibroblasts have been related to ribosome stalling during the elongation phase and low translation efficiency. Interestingly, erasing methylation from coding regions did not boost translation but notably reduced translation efficiency. A negative effect of m^6^A on elongation has also been reported in the breast cancer cell line MCF7. In this particular study, when investigating the link between transcription and translation the authors observed that suboptimal transcription rate leads to increased m^6^A content within the CDS, which ultimately results in a reduction of translation (80). In addition, it has been shown that m^6^A can also negatively modulate global translation by shifting m^6^A-marked mRNAs from polysomes to P-bodies through IGF2BP3. Lower global m^6^A levels are associated to an increased translation rate and decreased P-body enrichment, suggesting that the m^6^A-IGF2BP3 axis plays a crucial role in regulating the transition between translating and non-translating mRNA pools (81).

In contrast, the deposition of the m^6^A mark within the CDS may also enhance the translation of the target mRNAs. Indeed, it has been shown that m^6^A within the CDS contributes to the resolution of mRNA secondary structures and the elongation-promoting effect requires the RNA helicase containing m^6^A reader YTHDC2 (82). Furthermore, in acute myeloid leukemia (AML), METTL3, operating independently of METTL14, is recruited to chromatin by the CAATT-box binding protein CEBPZ and localizes to the transcription start site to regulate the translation of target genes. Genes bound by METTL3 are significantly enriched in [GAG]n sequences, which typically induce ribosomal arrest. However, m^6^A modification of these sequences alleviates ribosome stalling, indirectly facilitating the translation of these genes. Notably, METTL3 deletion does not affect the transcription of target genes such as SP1 and SP2. Instead, methylation loss causes a shift of transcripts to lower polysome fractions, thereby reducing protein synthesis and consequently impeding tumorigenesis of AML cells (83). Furthermore, in lung adenocarcinoma, the m^6^A mark within the CDS of the *FBXW7* mRNA has been shown to promote its translation. FBXW7 functions as a tumor suppressor gene by regulating the degradation of oncoproteins, such as c-MYC, suggesting a tumor-suppressive role of m^6^A specifically in this cellular context (84).

## Translation regulation of oncogenes and tumor suppressor genes

Boosting oncogene translation is a multifaceted process involving various regulatory events. This intricate orchestration includes changes in transcription, post-transcriptional modifications, and the dynamic interplay between RBPs, RNA modifications, and miRNA-mediated control, amongst others. Specifically, the deposition of the m^6^A mark on certain mRNAs has been largely associated with uncontrolled proliferation and survival of cancer cells (Table 1) (85–87). Among these, *MYC* is a prominent m^6^A-modified oncogene, characterized by a significant translation upregulation during tumorigenesis (88–91). It stimulates proliferation by activating several cyclins and CDK4, enabling the cell to enter the S phase, coordinating G_1_/S transition signaling, and enhancing metabolic activity for genomic replication (92). Several studies underscore the regulatory role of m^6^A in *MYC* expression during tumorigenesis. For instance, in gastric cancer, HBXIP is highly expressed and enhances METTL3 expression, which in turn leads to *MYC* mRNA methylation and subsequent translation, thereby promoting proliferation and cell migration (93). Similarly, in AML, elevated METTL3 induces the m^6^A methylation of *MYC*, *BCL2* and *PTEN* transcripts, enhancing their translation efficiency and resulting in cell survival, proliferation, and maintenance of the hematopoietic cell program (94). In triple-negative breast cancer, m^6^A sites within the 3′UTR of *MYC* mRNA promote its translation, activating splicing factors like SRSF11, which supports cell growth (95). Beyond direct associations with METTL3, other components of the m^6^A machinery, including ALKBH5, FTO, and METTL14, can modulate the level of the m^6^A mark at *MYC* mRNA. This impacts the recruitment of the reader YTHDF1 and subsequently affects *MYC* translation across various cancers (96–98).

**Table 1.** m^6^A -dependent translation regulation mechanisms

Cancer type	mRNA	m^6^A site	Reader	Role	Function	Ref.
Gastric cancer	*MYC*	CDS	-	Oncogenic	Cell proliferation, migration and invasion	(93)
AML	*MYC, BCL2, PTEN*	5′UTR, CDS, 3′UTR	-	Oncogenic	Survival and cell proliferation	(94)
TNBC	*MYC*	3′UTR	-	Oncogenic	Specific AS switches, tumorigenesis	(95)
B‐cell lymphoma	*SPI1, PHF12*	CDS	YTHDF3	Oncogenic	Tumor progression	(96)
LUAD	*MYC*	5′UTR, CDS, 3′UTR	YTHDF1	Oncogenic	Glycolisis and tumorigenesis	(97)
AML	*MYB, MYC*	5′UTR	eIF3	Oncogenic	Stem cell renewal and tumor growth	(98)
Melanoma	*EGFR*	3′UTR	-	Oncogenic	PLX4032 resistance	(99)
Breast cancer	*EGFR, TAZ*	3′UTR	-	Oncogenic	Cell proliferation	(100)
Gastric cancer	*CDCP1*	3′UTR	YTHDF1	Oncogenic	Tumor progression	(101,102)
Gastric cancer	*SPHK2*		YTHDF1	Oncogenic	Tumor progression	(103)
LUAD	*SLC7A1*	-	YTHDF1	Oncogenic	Tumor growth and ferroptosis	(104)
LUAD	*ENO1*	CDS	YTHDF1	Oncogenic	Glycolysis and tumor progression	(106)
OSCC	*BMI1*	3′UTR	IGF2BP1	Oncogenic	Tumor growth and metastasis	(107)
Ocular melanoma	*CTNNB1*	CDS	YTHDF3	Oncogenic	Tumor initiation and CSC properties	(108)
GIST	*MRP1*	5′UTR	YTHDF1, eIF-1	Oncogenic	Drug resistance	(109)
Bladder cancer	*ITGA6*	3′UTR	YTHDF1, YTHDF3	Oncogenic	Tumor adhesion, migration and invasion	(110)
Bladder cancer	*TROP2*	3′UTR	YTHDF1	Oncogenic	Tumor growth and metastasis	(111)
LUAD	*FBXW7*	CDS	-	Tumor suppressor	Tumor growth and apoptosis	(84)
PTC	*STEAP2*		YTHDF1	Tumor suppressor	EMT, tumor aggressiveness	(112)
Endometrial cancer	*PHLPP2*	-	-	Tumor suppressor	Tumor growth	(114)
Ocular melanoma	*HINT2*	3′UTR	YTHDF1	Tumor suppressor	Tumor growth, prognosis	(115)
Melanoma	*SPRED2*	CDS, 3′UTR	YTHDF1	Tumor suppressor	Tumor growth, metastasis	(116)

AML, acute myeloid leukemia; TNBC, triple-negative breast cancer; LUAD, lung adenocarcinoma; OSCC, oral squamous cell carcinoma; GIST, gastrointestinal stromal tumor; PTC, papillary thyroid carcinoma; EMT, epithelial–mesenchymal transition.

Other oncogenic pathways such as Wnt/β-catenin, PI3K/AKT/mTOR and RAS/RAF/MEK/ERK share similar regulation mechanisms. For example, in the A375R melanoma cell line, METTL3 promotes methylation of the epidermal growth factor receptor (*EGFR*), thereby enhancing its translation and facilitating the rebound activation of the ERK pathway, which is associated with chemoresistance (99). In breast cancer cells, peptidyl-prolyl *cis-*trans isomerase NIMA-interacting 1 (PIN1) interacts with METTL3 and prevents its ubiquitin-dependent proteasomal and lysosomal degradation. Specifically, PIN1-stabilized METTL3 is able to promote m^6^A-mediated translation of the transcriptional coactivator with PDZ-binding motif (*TAZ*) and *EGFR*, affecting cell proliferation and cell cycle (100). Furthermore, METTL3-mediated m^6^A-modification promotes *CDCP1* translation in gastric and bladder cancers (101). CDCP1 functions as a substrate of Src family kinases and regulates cell migration and extracellular matrix degradation during tumor progression and metastasis. Additionally, m^6^A deposition at the 3′UTR of *CDCP1* facilitates its translation and correlates with disease progression (102). Likewise, *SPHK2* transcript is also regulated by m^6^A through the same mechanism in gastric cancer, promoting tumorigenesis (103).

In lung adenocarcinoma, METTL3-mediated m^6^A modification enhances *SLC7A11* translation, fostering cell proliferation while suppressing cell ferroptosis (104), a nonapoptotic form of cell death known for selective elimination of tumor cells (105). Similarly, m^6^A-driven translation of *ENO1* transcripts stimulates glycolysis, contributing to the progression of lung adenocarcinoma (106). In oral squamous cell carcinoma (OSCC), m^6^A methylation of the 3′UTR region of the cancer stem cell marker *BMI1* enhancers *BMI1* translation by binding to IGF2BP1, leading to an increase in polysome-bound *BMI1* mRNA (107). In ocular melanoma, abnormal upregulation of YTHDF3 affects tumor initiation and progression by promoting *CTNNB1* translation. Methylation of the CDS region is essential for YTHDF3 to promote *CTNNB1* translation, which ultimately leads to the inactivation of Wnt/β-catenin targets and the alteration of stem cell-like properties (108). Additionally, m^6^A modification in the 5′UTR of *MRP1*, which encodes a multidrug transporter, facilitates *MRP1* mRNA translation via YTHDF1 and eEF-1 interaction, increasing drug resistance in gastrointestinal stromal tumors (109). Furthermore, m^6^A also regulates the adhesion, migration and invasion of bladder cancer cells by mediating the translation of the *ITGA6* transcript (110).

In addition, the interplay between different RNA modifications has also been linked to translation. For example, the dual deposition of m^6^A and m^7^G modifications promotes the translation of trophoblast cell surface protein 2 (TROP2) in bladder cancer. TROP2 is a cell surface glycoprotein that belongs to the EGFR family and is primarily involved in cell adhesion, signal transduction, and cell proliferation. METTL1 facilitates m^7^G deposition on specific tRNAs, while METTL3 synergistically methylates the *TROP2* transcript, thereby fostering TROP2 expression and promoting cell proliferation, migration, and invasion (111).

In contrast, fewer studies have highlighted the potential of m^6^A in contributing to the translation of tumor suppressors, both preventing and inhibiting tumorigenesis. For example, in lung adenocarcinoma, METTL3-dependent m^6^A modification in the CDS of *FBXW7* mRNA promotes its translation (84). FBXW7 plays a pivotal role as a tumor suppressor by orchestrating the proteasome-driven degradation of oncoproteins, including but not limited to c-MYC, MCL1, Notch1/4, cyclin E and mTOR. Similarly, in papillary thyroid cancer, m^6^A enhances the translation of the tumor suppressor *STEAP2* in a YTHDF1-dependent manner, leading to the inactivation of the Hedgehog signaling pathway and the epithelium to mesenchymal transition (EMT), restraining the aggressive tumor phenotype (112). In endometrial cancer, m^6^A regulates the PI3K/AKT pathway by promoting the YTHDF1-mediated translation of the phosphatase PHLPP2. The PHLPP family of proteins are potent tumor suppressors in many human cancers (113). This, alongside m^6^A-mediated mTORC2 degradation, contributes to AKT inhibition by maintaining its dephosphorylated state at serine 473 (114). Moreover, METTL3-mediated methylation of *HINT2* mRNA at 3′ and 5′UTR regions, known for its tumor suppressor functions, facilitates the binding of YTHDF1 to promote its translation (115). In addition, m^6^A also modulates the translation of specific transcripts in macrophages and tumor-associated macrophages. Tumors shape their microenvironment to promote tumor growth by recruiting stromal cells such as tumor-associated macrophages, which are associated with a poor patient prognosis. In this cellular context, m^6^A controls *SPRED2* translation, a negative regulator of the ERK signaling pathway. Reduced m^6^A levels hinder YTHDF1-mediated translation of *SPRED2*, contributing to tumor growth and metastasis. This suggests that METTL3 has the potential to modulate the tumor microenvironment by regulating the translation of key tumor suppressor transcripts (116).

In summary, these findings underscore the intricacy of the m^6^A modification. It serves not only as an epitranscriptomic mark promoting oncogenic processes but also as a potent tumor suppressor. This complexity is compounded by its dependency on the specific cell type or cancer context, with limited concrete evidence or common mechanistic pathways identified thus far.

## Translation regulation mediated by cytoplasmic METTL3

**Figure 5.**
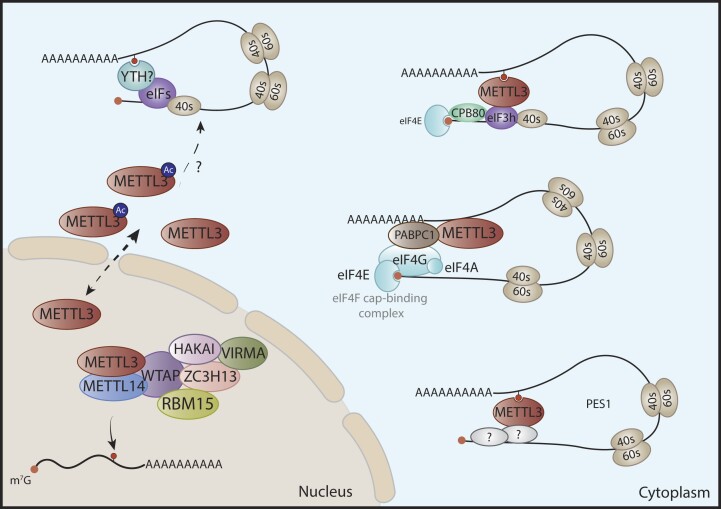
Translation regulation mediated by cytoplasmic METTL3. Schematic representation of the mechanisms by which METTL3 in the cytoplasm enhances the translation of specific mRNAs in cancer cells. In lung cancer, METTL3 has been described to act as an m^6^A reader in the 3′UTR of oncogenic transcripts, enhancing their translation through direct interaction with eIF3h. In gastric cancer, independent of m^6^A, METTL3 binds PABPC1 to facilitate its interaction with the cap-binding complex and promote the loop configuration, ultimately promoting gastric tumorigenesis. Cytoplasmic METTL3 has been suggested to promote the translation of *PES1*, independent of m^6^A, in chronic myeloid leukemia, but possible interacting partners and specific mechanisms have not been determined. Acetylation of METTL3 mediates its cytoplasmic localization in breast cancer.

The canonical role of METTL3 involves its function as an m^6^A methyltransferase within the nuclei, acting in a co-transcriptional manner. Yet, recent discoveries have revealed that METTL3 can undergo mislocalization to the cytoplasm of cancer cells, where it boosts the translation of particular oncogenes (Figure 5).

In 2016, Lin *et al.* demonstrated that silencing METTL3 in lung cancer cells had minimal impact on mRNA levels but drastically reduced the protein levels of several oncoproteins, including EGFR and TAZ. METTL3 depletion completely altered the polysome fractions, with depleted cells showing a decrease in the 80S peak. m^6^A target genes presented a shift to sub-polysome fractions compared to scrambled control cells, suggesting that METTL3 regulates the translation of a subset of m^6^A-marked mRNAs. Interestingly, this cytoplasmic translation-regulation role of METTL3 operates independently of the YTHDF1 reader. Mechanistically, METTL3 acts as a 3′UTR m^6^A reader, associating with eIF3, and indirectly interacting with CBP80 and eIF4E, thereby promoting mRNA translation independently of its methyltransferase activity (117).

Subsequent studies have shown that METTL3 can associate with polyribosomes and localize in close proximity to cap-binding proteins such as CBP80 and eIF4E, thereby enhancing the looping of mRNAs through its direct interaction with eIF3h (118). These findings indicate that METTL3 preferentially affects the translation of transcripts featuring longer 3′UTRs associated with cellular progression and apoptosis processes. In particular, the 150–200 amino acid domain within METTL3, containing a putative alpha-helix domain that is highly conserved in mammals, is essential for the METTL3-eIF3h interaction. Specifically, a conserved alanine at position 155 is pivotal for this interaction, which in turn facilitates translation and ribosome recycling. The A155P METTL3 mutant retains association with METTL14 and can catalyze m^6^A deposition but exhibits severely impaired translation ability of key oncoproteins such as BRD4 and EGFR.

Although context-specific, it has been shown that METTL3 can also promote translation in an m^6^A-independent manner. Thus, METTL3 associates with PABPC1, enhancing its interaction with the eIF4F complex, and improving RNA loop configuration (119). In gastric cancer, <5% of total METTL3-bound transcripts possess m^6^A sites, demonstrating that METTL3 preferentially binds to non-m^6^A methylated mRNAs. This implies that METTL3’s role as a translational promoter is primarily due to its direct interaction with PABPC1, and that the propensity to bind mRNA may be explained by mechanisms beyond m^6^A modification. Investigating the molecular mechanisms influencing the preference of METTL3 for non-modified over m^6^A-methylated mRNAs warrants further exploration. Interestingly, cytoplasmic and catalytically inactive METTL3 was able to induce a global activation of translation and was sufficient to rescue the invasive and proliferative phenotype of gastric cancer cells upon knockdown of endogenous METTL3.

Likewise, in ovarian carcinoma, cytoplasmic METTL3 promotes AXL translation, contributing to the initiation and progression of ovarian cancer (120). The cytoplasmic localization of METTL3 is critical in controlling mRNA translation, promoting EMT and tumorigenesis through AXL regulation. However, this particular study did not perform any translation experiments to confirm whether AXL translation is directly regulated by METTL3 or if the observed effect is due to an indirect effect. Therefore, further investigation is needed to better understand the molecular mechanism behind their strong correlation. Similarly, in AML, METTL3 regulates WTAP (121). Notably, although *WTAP* mRNA levels are low despite high protein levels in AML, both wildtype and catalytically inactive METTL3 are able to bind *WTAP* mRNA in the cytoplasm, promoting its translation. METTL3, similar to eIF3, is associated to active translating ribosome and its overexpression results in increased *WTAP* mRNA levels in polysome fractions. In another study, the translation of Pescadillo 1 (*PES1*), an essential regulator of ribosome biogenesis and cell proliferation, has been reported to be modulated by cytoplasmic METTL3 independently of its catalytic activity in chronic myelogenous leukemia cells. However, this study lacks experimental validation, such as the mutation of the nuclear localization signal of METTL3, to ensure that the cytoplasmic localization of METTL3 is directly responsible for the translation of PES1 (122).

The mechanism behind the cytoplasmic localization of METTL3 remains elusive. PTMs can modulate the subcellular localization of various signaling molecules (123), and METTL3 itself undergoes SUMOylation, methylation, ubiquitination, phosphorylation and acetylation (124–127), being the latest one associated with nuclear-to-cytoplasmic translocation of METTL3 in cancer metastasis (128). In contrast to previous findings where cytoplasmic METTL3 displayed an oncogenic role, this investigation reveals a strong correlation between nuclear METTL3 levels and node breast cancer metastasis, being the cytoplasmic expression restricted to normal and non-invasive tissues. Mechanistically, the acetylation of lysine at position 177 (K177) within the NLS of METTL3 disrupts its interaction with importin α5, abrogating the nuclear translocation. In addition, K177 acetylation impedes m^6^A deposition in the cytosol, facilitating protein translation by enhancing the interaction of METTL3 with CBP80, eIF3b and eIF3h. Intriguingly, the study unveiled a positive feedback loop regulated by IL-6, which is an m^6^A target. Essentially, IL-6 promotes SIRT1-mediated deacetylation of METTL3 and nuclear translocation, thus increasing global m^6^A levels and promoting the stability of oncogenic mRNAs. Notably, SIRT1 inhibition reverses the deacetylation nuclear shift, leading to low m^6^A levels and decreased lung metastasis in murine models.

## Perspectives

Since the development of high-throughput methods for m^6^A mapping in 2012, the number of studies focused on elucidating the role of this RNA modification in cancer has dramatically increased. From mediating mRNA degradation to promoting mRNA stability and translation, the impact of m^6^A spans throughout the mRNA lifecycle. While much work remains to be done, the accumulating evidence suggests that the effect of m^6^A on a specific transcript is very context-dependent. m^6^A deposition occurs within a consensus motif (DRACH; D = A/G/U, R = A/G, H = A/U/C), which is widely prevalent in the transcriptome. However, a major feature of m^6^A distribution, not solely explained by sequence characteristics, is the marked enrichment of m^6^A in long internal exons and near stop codons. In other words, not all the instances of the DRACH motif harbor the m^6^A modification. For instance, the secondary structure of the mRNA molecule can influence the accessibility of the methyltransferase complex to the motif. More exposed motifs are more likely to be m^6^A modified. Additionally, it has recently been suggested that sites containing an m^6^A consensus sequence are methylated by default unless they fall within a window of approximately ∼100 nucleotides from a splice junction, where their modification is inhibited by the exon junction complex (129–131). Moreover, within the context of translation, as highlighted in this review, the distinct locations of m^6^A within a given mRNA have been associated with differential effects, adding an extra layer of complexity to unravelling its specific functions. Finally, it should be noted that most of the studies included in this review focused on m^6^A as a single modification on the mRNA. However, an mRNA may simultaneously bear more than one m^6^A mark and/or different RNA modifications. Therefore, utilizing mapping tools that allow simultaneous mapping of different modifications could provide valuable insights into the potential interplay of various chemical marks in translation (132,133).

m^6^A exhibits a dual role as both an oncogene and a tumor suppressor mark. As an oncogenic chemical modification, it enhances tumorigenesis by promoting the translation of specific oncogenes, such as MYC. Conversely, m^6^A can be deposited in tumor suppressor transcripts, thereby inhibiting tumor growth and cancer progression. Mechanistically, the deposition of the m^6^A mark in the target mRNA may increase its translation efficiency, the polysome-bound mRNA or facilitate the recognition of a reader protein. Ultimately, these processes culminate in the recruitment of the translation machinery and the promotion of protein synthesis.

The use of METTL3 inhibitors may be a promising strategy for counteracting the pro-oncogenic effects of m^6^A. Small molecules such as STM2457, UZH1a and UZH2 act as METTL3 inhibitors by competing with the endogenous cofactor SAM, thereby interfering with the methyltransferase activity, and leading to a decrease in m^6^A levels (134–136). Notably, the pharmacological inhibition of METTL3 is currently undergoing clinical trials in subjects with advanced malignancies, led by STORM Therapeutics. In addition to SAM-competitive inhibitors, inhibitors targeting the interaction between METTL3 and METTL14 have also been developed. These inhibitors, such as Eltrombopag and CDIBA-4, bind directly the METTL3–METTL14 heterodimer, impacting its catalytic activity, which correlates with severely impaired proliferation in MOLM-13 cells and other leukemia cell lines (137,138).

The synergistic action of METTL3 inhibitors and translation inhibitors holds potential for developing effective therapeutic approaches against tumors characterized by oncogenic m^6^A marks and pronounced translation dysregulation. Among the most commonly used translation inhibitors are those that target mTOR, one of the main regulators of translation (139), Silvesterol, which targets eIF4A (140), and Omacetaxine that binds to the A-site of the ribosome blocking the elongation step (141). This combination approach could provide a potential avenue for innovative and impactful cancer therapies. While the significance of METTL3 inhibitors is unarguable, the methyltransferase activity of METTL3 appears to be dispensable for its translation role in specific cancers, such as lung and gastric cancer. Consequently, new efforts have focused on developing small molecules that specifically bind to METTL3, aiming to trigger its degradation through approaches like PROTAC-based degradation (142). A novel degrader of the METTL3-METTL14 complex, WD6305, has emerged (143,144). Although WD6305 directly interacts with METTL3, it also facilitates the degradation of METTL14. This indirect degradation results from disruption of the integrity of the complex, consistent with previous findings that METTL14 stability is dependent on METTL3 (144). This approach offers a more targeted and potentially effective strategy for treating cancers where cytoplasmic METTL3 enhances oncogenic translation without relying on its enzymatic activity.

The discovery that cytoplasmic METTL3 directly regulates translation independent of its methyltransferase activity represents a significant milestone in the field. The identification of the residue involved in translation modulation, exemplified by the A155P mutant, along with investigations involving the catalytically impaired METTL3 have proven crucial in determining whether the translation-promoting mechanisms of METTL3 are m^6^A-dependent or independent. Despite these advancements, some of the studies reported here lack translation-related validation, relying solely on protein levels measurements. Although it is undeniable that cytoplasmic METTL3 can impact translation and contribute to tumorigenesis, this mechanism appears highly context-dependent, with a limited number of published studies to date. Further research is imperative to validate current results and gain insights into the mechanisms governing METTL3 translocation to the cytoplasm of cancer cells and whether similar mechanisms also operate in normal cells.

## Data Availability

No new data were generated or analysed in support of this research.
